# Effects of therapeutic plasma exchange (TPE) on efficacy and adverse events in patients with autoimmune neurological diseases: a systematic review and meta-analysis based on real-world studies and single-arm clinical trials

**DOI:** 10.3389/fneur.2026.1916922

**Published:** 2026-08-26

**Authors:** Jinyu Tao, Xiaoqin Yang, Han Tang, Jialin He, Siyin Gong, Jinfang Li

**Affiliations:** Department of Neurology, The Second Affiliated Hospital of Chongqing Medical University, Chongqing, China

**Keywords:** adverse events, efficacy, meta-analysis, neuro-immunological disease, therapeutic plasma exchange

## Abstract

**Background:**

Therapeutic plasma exchange (TPE) is a core intervention for autoimmune neurological diseases (ANDs). To date, clinical efficacy and safety of TPE across disease subtypes, age strata and regions have not been systematically synthesized. This meta-analysis aimed to evaluate the clinical response, mortality and adverse event (AE) rates of TPE in patients with ANDs.

**Methods:**

Relevant real-world observational studies and single-arm trials were retrieved from PubMed, Embase and Cochrane Library. We conducted single-arm meta-analysis to pool effect sizes with 95% confidence intervals (CIs). Subgroup and leave-one-out sensitivity analyses were used to assess heterogeneity and result stability.

**Results:**

In total, 38 studies comprising 2,199 patients were included. Response rates differed significantly across study-level age strata, whereas differences across disease and regional subgroups were not statistically significant. The all-cause in-hospital mortality rate among patients undergoing TPE was 4% (95% CI: 3–5%, *I*^2^ = 36.5%), and this value does not represent mortality attributable to TPE. A total of 49 fatal cases were reported. The most common adverse events were hypotension (12%), coagulation disorders (11%), catheter-related infections (8%), allergic reactions (7%), and hypocalcemia (7%). Some studies reported adverse events as per the number of patients and others as per TPE sessions. This study calculated the incidence rates of adverse events based on the number of patients.

**Conclusion:**

Patients with ANDs show a relatively high rate of clinical improvement after TPE. Most deaths are attributable to underlying disease progression rather than TPE itself. Further high-quality multicenter studies are required to optimize patient selection and treatment protocols for TPE.

## 1 Introduction

### 1.1 Background

Autoimmune neurological diseases (ANDs) are a group of disorders in which abnormal immune responses involve the central or peripheral nervous system. Their core feature is that the immune system aberrantly attacks components of the nervous system, and this process can trigger inflammation, demyelination, and neuronal damage, leading to various neurological symptoms (1). These diseases are important causes of acquired neurological deficits, limb paralysis, respiratory failure, and even death in middle-aged and young populations. In recent years, with improvements in disease recognition and advancements in immunological research, the diagnostic spectrum of ANDs has expanded to include Guillain-Barre syndrome (GBS), myasthenia gravis (MG), neuromyelitis optica spectrum disorder (NMOSD), autoimmune encephalitis (AE), and chronic inflammatory demyelinating polyneuropathy (CIDP). The pathogenesis may involve autoantibody-mediated mechanisms, cellular immune abnormalities, complement activation, release of inflammatory cytokines, and breakdown of immune tolerance (2).

Therapeutic plasma exchange (TPE) is an important immunomodulatory intervention for various antibody-mediated or immune-mediated neurological diseases. As a blood purification technique that separates and replaces plasma by centrifugation or membrane filtration (3), TPE can remove pathogenic autoantibodies, immune complexes, complement components, and inflammation-related mediators from the circulation (4, 5), thereby relieving symptoms and controlling conditions in some neuro-immunological disorders. In clinical practice, TPE, together with intravenous immunoglobulin (IVIG), corticosteroids, and other immunotherapies, constitutes an important treatment strategy for ANDs. However, its appropriate indications and strength of evidence vary by disease type and stage (6, 7). According to relevant guidelines, TPE has been recommended for specific clinical scenarios, like first-line treatment for GBS and short-term treatment for crisis or acute exacerbations of MG (8). Nevertheless, the real-world clinical response and safety of TPE may still be influenced by factors like baseline disease status, timing of treatment, exchange protocol, combined treatment, and procedural management across different disease subtypes, age groups, and regional healthcare settings. In particular, evidence on TPE remains relatively limited in children, old patients, and rare ANDs (9).

Although TPE is widely used in clinical practice, the currently available systematically integrated evidence regarding its real-world efficacy and safety remains relatively limited. Most existing studies are single-center retrospective or single-arm studies, and considerable heterogeneity was found across studies in terms of disease spectrum, patient age, timing of treatment, exchange protocol, and methods of reporting adverse events. In particular, systematic and comprehensive evaluations are still lacking regarding the efficacy across different disease subtypes, age groups, and populations from different geographic regions, as well as the incidence of common adverse events and death outcomes. Thus, the present study intended to systematically include globally published real-world observational studies and eligible single-arm clinical studies, conduct a systematic review and meta-analysis, evaluate the clinical response rate, mortality rate, and characteristics of adverse events in individuals with ANDs treated with TPE, and further ascertain potential differences through subgroup analyses by disease type, age, and geographic region, thereby providing evidence for the individualized and standardized application of TPE in clinical practice.

## 2 Methods

This meta-analysis was implemented as per the Preferred Reporting Items for Systematic Reviews and Meta-Analyses (PRISMA) guidelines. The protocol was registered with the International Prospective Register of Systematic Reviews (PROSPERO) (registration number: CRD420250652869).

*Literature search strategy*: PubMed, Embase, and Cochrane Library were retrieved. The initial search covered the period from January 1, 2000 to March 3, 2025. A supplementary search was subsequently performed on January 6, 2026 to include relevant studies published between March 4, 2025 and January 6, 2026, using the same search strategy and databases as the initial search, with the language restricted to English. The search strategy combined medical subject heading (MeSH) and free-text terms, primarily focusing on terms related to TPE and ANDs. Terms related to TPE included “therapeutic plasma exchange,” “plasma exchange,” “plasmapheresis,” and “plasma exchange therapy.” Terms related to ANDs encompassed “autoimmune neurological disease,” “Guillain-Barré syndrome,” “myasthenia gravis,” “neuromyelitis optica spectrum disorder,” “autoimmune encephalitis,” “chronic inflammatory demyelinating polyneuropathy,” and “stiff-person syndrome.” Study types were determined during the screening process based on the inclusion and exclusion criteria. The complete search strategy is provided in Supplementary Table S1.

### 2.1 Study selection

Studies that satisfied conditions below were included: (1) Study population: individuals diagnosed with ANDs (e.g., GBS, NMOSD, MG, and stiff-person syndrome (SPS)). (2) Intervention: individuals treated with TPE. (3) Outcome measures: clinical efficacy and adverse events (e.g., hypotension, infection). (4) Study design: real-world observational studies primarily included and single-arm clinical trials additionally incorporated to supplement the evidence on the efficacy and safety of single-arm interventions.

Studies meeting conditions below were excluded: (1) Duplicate publications. (2) Non-original studies (e.g., conference abstracts), animal studies, reviews, guidelines, etc. (3) Studies published before 2000 (because early TPE techniques and peri-procedural management differed considerably from modern practice). (4) Studies that did not report the required outcome measures. (5) Studies for which the full English texts could not be obtained.

Literature screening was performed independently by two investigators (JYT and XQY), and a third reviewer (HT) consolidated the screening results.

### 2.2 Data extraction

Data were independently extracted by two investigators (JT and XY), and divergences were addressed by a third reviewer (HT). The following data were acquired from each eligible study: first author, year of study, sample size, geographic region, age, sex composition, disease duration, intervention measures, evaluation criteria for efficacy, definitions of response (Supplementary Table S2), patient response rate, mortality rate, and types of adverse events (encompassing fever and chills, dyspnea, allergic reactions, hypocalcemia, arrhythmia, nausea and vomiting, hypertension, hypotension, catheter-related infections, deep vein thrombosis, and coagulation disorders) as well as the incidence rates of adverse events.

### 2.3 Risk of bias assessment

The methodological quality of the included studies was evaluated utilizing the Methodological Index for Non-Randomized Studies (MINORS) scale (10). Since most of the included studies were non-comparative, the first eight items of the MINORS scale applicable to non-comparative studies were adopted for scoring, including research with a defined purpose, patient consistency of inclusion, anticipated data collection, outcome indicator response study purpose, objectivity of outcome indicators, adequate follow-up time, lost visit rate below 5%, and estimated sample size. Each item was scored from 0 to 2: 0 for not reported, 1 for reported but inadequate, and 2 for reported and adequate. The total score ranged from 0 to 16. Quality assessment was performed independently by two investigators, and divergences were addressed through discussion. A third investigator was consulted when necessary. The results of the quality assessment are presented as total scores and item scores for each study.

### 2.4 Data synthesis

The primary outcome of the present study was the response rate in disease subgroups, and the secondary outcomes were the mortality rate and the incidence rate of adverse events. Statistical analyses were implemented utilizing R 4.3.3 and RStudio. For single-arm proportional data, a single-arm rate meta-analysis was adopted to pool the response rate in disease subgroups, mortality rate, and the incidence rate of adverse events, and the pooled rates with their 95% confidence intervals (CI) were calculated.

Heterogeneity across studies was appraised utilizing Cochran’s *Q* test and the *I*^2^ statistic. For *I*^2^ > 50% or the *Q* test *p* < 0.10, substantial heterogeneity was considered, and a random-effects model was preferentially adopted. When heterogeneity was low, a fixed-effects model was applied. Given the clinical heterogeneity among the included studies in terms of disease type, age structure, TPE protocol, and definition of outcomes, a random-effects model was preferentially used for the primary outcome and most safety outcomes.

For the primary outcome, subgroup analyses were carried out by disease type, age group, and study region to ascertain potential sources of heterogeneity. Disease subgroups included GBS, MG, AE, and SPS. Age subgroups were classified as <18 years, 18–60 years, and >60 years according to the mean or median ages in the original studies. Region subgroups were categorized as Asia, Europe, the Americas, and the Middle East based on the study location. Studies that included multiple diseases but did not report disease-specific efficacy separately were included only in the overall response rate analysis and not in the disease subgroup analyses.

For adverse events, the incidence rates of adverse events based on the number of patients and TPE sessions were separately extracted according to the reporting methods of the original studies. The primary results of this study were reported using the incidence rates of adverse events based on the number of patients. For rare adverse events or zero-event studies, continuity correction or appropriate transformation methods applicable to single-arm proportional data were adopted for pooled analysis.

A leave-one-out sensitivity analysis was implemented to appraise the robustness of the primary outcome and key safety outcomes. When at least 10 studies were included for a given outcome, funnel plots and Egger’s test were used to evaluate potential publication bias. If publication bias was suggested, trim-and-fill analysis was further performed to evaluate its impact on the pooled results. All statistical tests were two-sided, and *p* < 0.05 was considered statistically significant.

## 3 Results

### 3.1 Study selection

**Figure 1:**
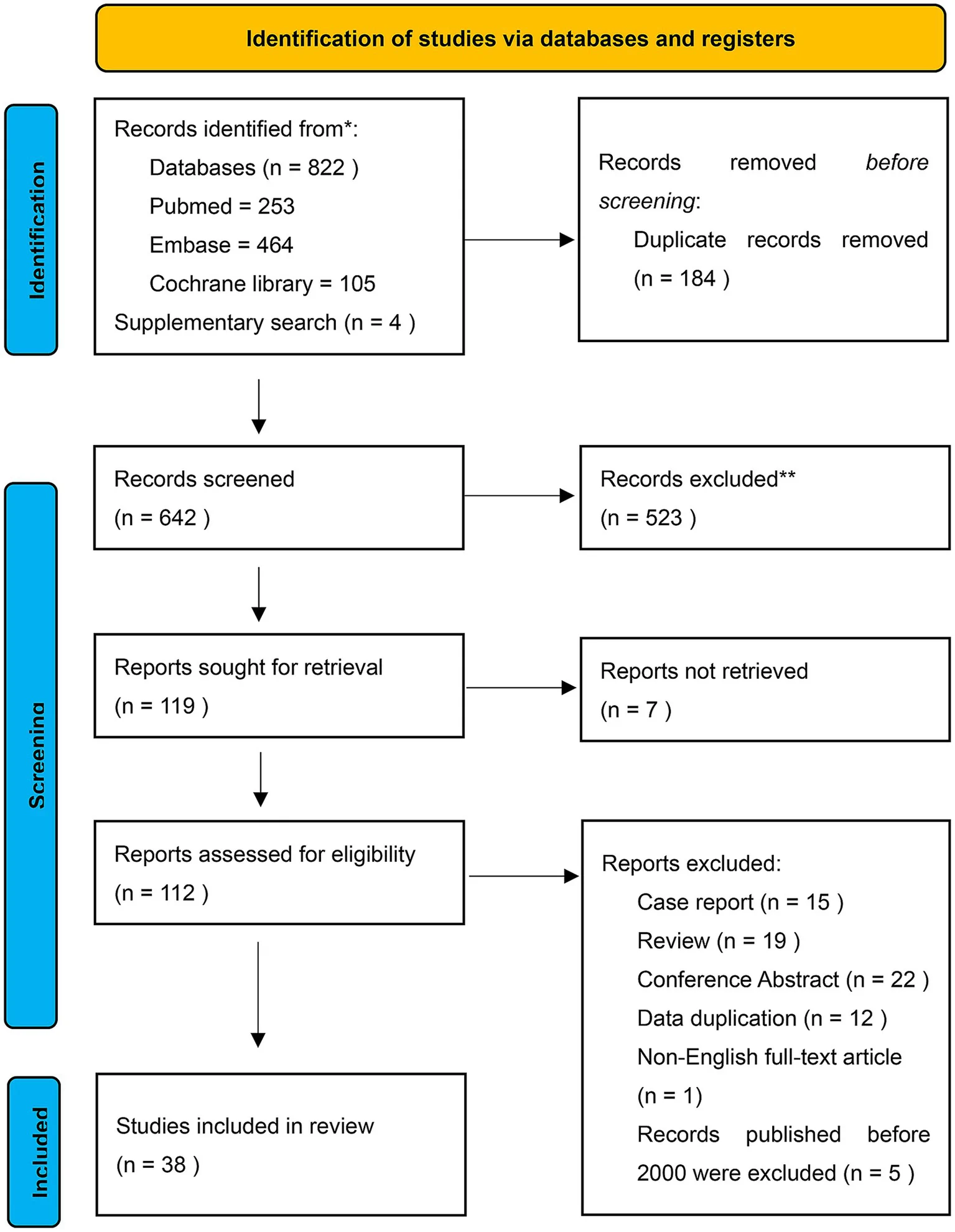
Methodology process for literature inclusion.

The initial search yielded 822 records, including 253 from PubMed, 464 from Embase, and 105 from Cochrane Library. After automatic duplicate checking using EndNote and manual verification, 184 duplicate records were removed, and 638 records were retained for title and abstract screening. Following title and abstract screening, 523 records that were not directly relevant to the research topic were excluded. The full texts of the remaining 115 articles were then assessed. Among these, 7 articles were excluded because the full texts could not be obtained, and another 74 articles were eliminated due to reasons such as inappropriate study design, language restriction, publication year before 2000, or failure to meet the inclusion criteria regarding study population or outcome measures. We ultimately included 34 studies during the initial search. During the supplementary search, additional 4 eligible studies were incorporated. Thus, a total of 38 studies were included finally (3, 11–47). The literature screening process is shown in Figure 1.

### 3.2 Study characteristics and quality assessment

As shown in Tables 1, 38 studies were included in this study. All of these were retrospective cohort studies or single-arm clinical trials of non-randomized controlled trials, comprising 2,199 individuals. The age ranged from 8 months to 94 years, and regions included Europe, the Americas, Asia, and the Middle East. These studies mainly involved neuro-immunological diseases like GBS, MG, CIDP, SPS, NMOSD, multiple sclerosis, and AE. The main adverse events encompassed fever and chills, dyspnea, allergic reactions, hypocalcemia, arrhythmia, nausea and vomiting, hypertension, hypotension, catheter-related infections, deep vein thrombosis, and coagulation disorders. The primary outcome measures included the response rate in disease subgroups, mortality rate, and incidence rate of adverse events in individuals after receiving TPE.

**Table 1:** Characteristics of studies included.

Study	Sample size	Age	Gender (male/female)	Country	Number of plasma exchange sessions	Type of replacement fluid	Prior use of corticosteroids/immunoglobulins/immunosuppressants before TPE	Number of responders	Number of deaths	Disease type
Aljezani et al. 2024 (39)	15	13*	12/3	Saudi Arabia	5.3	5%HA + NS	Partial	12	1	GBS: 7
										MG: 3
										TM: 2
										NMOSD: 2
										SLE-E: 1
Ali et al. 2024 (11)	36	8.3 ± 2.9	20/16	Damascus, Syria	3.89	4%HA + NS	Partial	35	1	GBS: 36
						FFP#				
Błasiak et al. 2025 (12)	81	13.8*	—	Cracow, Poland	4.44	FFP/HA	Not reported	57	1	GBS: 65
										MG: 8
										MS: 3
										ADEM: 2
										MMN: 3
Bava et al. 2024 (13)	99	36*	60/39	India	4.24	FFP/FFP + 5%HA	Not reported	67	9	GBS: 50
										AE: 14
										MG: 12
										NMOSD: 8
										CIDP: 5
										Others: 10
Carandina-Maffeis et al. 2004 (14)	26	28	7/19	Brazil	6.58	4%HA + NS	Yes	26	1	MG: 26
Chen et al. 2024 (15)	30	47.23 ± 7.99	21/9	China	3	4%HA	No	20	0	MG: 30
Dogra et al. 2020 (16)	18	35.5	5/13	India	4.2 ± 1.2	4%HA + FFP + NS	Not reported	18	1	MG: 18
Dada and Kaplan 2004 (17)	10	—	—	United States	5.3	5% HA	Partial	8	0	GBS: 10
						FFP#				
Gafoor et al. 2015 (40)	230	42.3	131/99	India	4.3	FFP	Partial	/	6	GBS: 203
										MG: 8
										CIDP: 11
										NMOSD: 4
										ADEM: 4
Gala-Błądzińska et al. 2020 (18)	91	53.9	58/33	Poland	4.5	HA + FFP + NS	Partial	/	3	GBS: 36
										CIDP: 19
										MG: 16
										ADEM: 7
										MS: 10
										NMOSD: 3
Kaya et al. 2013 (19)	115	50*	53/62	Türkiye	6.7	5%HA/FFP	Partial	103	0	GBS: 67
										ADEM: 20
										CIDP: 12
										MS: 8
										MG: 7
										Others: 1
Kaynar et al. 2008 (41)	57	49*	42/15	Türkiye	5.2	HA/FFP	Partial	47	0	GBS: 41
										MG: 11
										ADEM: 3
										CIDP: 1
										MS: 1
Korkmaz et al. 2025 (42)	240	50 ± 18.1	—	Türkiye	5*	HA/FFP/HA + FFP	Partial	195	0	GBS: 118
										MG: 68
										MS: 50
										PNS: 5
										CIDP: 4
										NMOSD: 4
										VGKC-Ab diseases: 1
										MOGAD: 3
										ADEM: 1
										ON: 4
										TM: 2
Kumar et al. 2015 (20)	35	32	11/24	India	4.2 ± 1.2	HA + FFP + NS	Yes	28	3	MG: 35
Kumawat et al. 2024 (21)	5	47	4/1	India	8.6	FFP + NS + HES	Yes	4	1	SPS: 5
Lehmann et al. 2008 (22)	7	64*	3/4	Germany	5*	HA + NS	Yes	2	0	MMN: 7
Mercure-Corriveau et al. 2023 (44)	39	48 ± 14	9/30	United States	5*	HA + NS	Yes	23	0	SPS: 39
Moser et al. 2019 (3)	40	41	14/26	Austria	5.6	NR	Yes	30	0	MS: 21
										AE: 12
										SLE-E: 3
										ON: 2
										ADEM: 1
										NMOSD: 1
Naik et al. 2021 (45)	4	11.5	1/3	India	5	FFP + NS/HA	Yes	2	1	Anti-NMDAR encephalitis: 4
Nieto-Aristizábal et al. 2020 (46)	187	50*	83/104	Colombia	5*	HA/SG/HA + gelatin/HA + FFP	Yes	99	3	GBS: 53
										MG: 70
										NMOSD: 35
										CIDP: 23
										AE: 6
Pagano et al. 2014 (47)	9	55	2/7	United States	5	5%HA + NS	Yes	7	0	SPS: 9
Seyhanli et al. 2022 (23)	207	51*	123/84	Türkiye	6*	FFP/HA	Yes	207	0	GBS: 26
										MG: 72
										CIDP: 14
										MS and ADEM: 65
										Others: 30
Shah et al. 2020 (24)	32	12.5*	11/21	United States	5.8	5%HA/5%HA + FFP	Yes	24	0	AE: 18
										ADEM: 5
										NMOSD: 5
										TM: 4
Sheckley et al. 2021 (25)	14	61.5 ± 14.3	6/8	United States	5	5%HA	Yes	14	0	MG: 14
						FFP#				
Sinanović et al. 2017 (26)	77	51 ± 15.9	42/35	Bosnia and Herzegovina	3	NR	Yes	67	3	GBS: 29
										MG: 8
										CIDP: 18
										MS: 9
										NMOSD: 3
										Others: 10
Tombak et al. 2016 (27)	63	48*	37/26	Türkiye	82	5%HA/HES/FFP	Yes	51	2	GBS: 22
										MG: 21
										CIDP: 7
										MS: 2
										NMOSD: 2
										ADEM: 2
										TM: 1
										Others: 6
Yamada et al. 2015 (28)	3	—	—	United States	Not reported	5%HA	Yes	3	0	MG: 3
Yıldırım et al. 2021 (29)	23	9.2 ± 4.8	14/9	Türkiye	8.1 ± 4.4	FFP	Yes	19	5	GBS: 8
										AE: 5
										MG: 1
										TM: 1
										ADEM: 3
										Others: 5
Zhang et al. 2021 (30)	33	—	—	China	5	5%HA + NS + plasma	Yes	31	0	Anti-NMDAR encephalitis: 30
										Anti-GABAbR encephalitis: 2
										Anti-LGI1 encephalitis: 1
Totzeck et al. 2022 (31)	20	65.5	11/9	Germany	5	4%HA	Yes	12	0	MG: 20
Fateen et al. 2023 (43)	24	7.58 ± 2.04	11/13	Pakistan	5.2	Not reported	Not reported	—	0	GBS: 16
										AE: 2
										CIDP: 1
										TM: 1
										NMOSD: 4
Olsen et al. 2021 (32)	17	57.5	5/12	United States	9	5%HA	Not reported	12	1	TS-HDS neuropathy: 17
Codron et al. 2017 (33)	30	65 ± 14	25/5	France	21.5*	4%HA	Yes	18	0	CDPN: 30
Vikrant et al. 2017 (34)	31	45.8 ± 18.3	21/10	India	3.8 ± 1.5	4%HA + NS + FFP	Partial	24	1	GBS: 31
Pham et al. 2025 (35)	36	7.4*	14/22	Vietnam	5*	FFP/5%HA/FFP + 5%HA	Yes	25	3	AE: 36
Jamal et al. 2025 (36)	109	54.4	45/64	United States	5	5%HA/FFP	Yes	—	0	MG: 35
										AIDP/CIDP: 14
										NMO/MOGAD: 14
										AE: 14
										GBS: 11
										Others: 21
Gupta et al. 2025 (37)	53	48*	38/15	India	5*	4-5%HA	Yes	46	0	GBS: 18
										MG: 11
										NMOSD: 6
										CIDP: 5
										AE: 6
										TM: 6
										MS: 1
Eichinger et al. 2025 (38)	53	13*	21/32	Austria	7*	Not reported	Partial	38	0	MS: 12
										MG: 2
										AE: 10
										GBS: 5
										TM: 5
										ADEM: 4
										NMOSD: 3
										Others: 12

*Median age. ^#^Additional fresh frozen plasma (FFP) was infused to supplement coagulation factors only when the patient had a coagulation factor deficiency. —: or. Baseline: before TPE treatment. FFP, fresh frozen plasma; HA:, human albumin; IVIG, intravenous immunoglobulin; HES, hydroxyethyl starch; SG, succinylated gelatin; GBS, Guillain-Barre syndrome; MG, myasthenia gravis; MS, multiple sclerosis; NMOSD, neuromyelitis optica spectrum disorder; CIDP, chronic inflammatory demyelinating polyneuropathy; AIDP, acute inflammatory demyelinating polyneuropathy (subtype of GBS); ADEM, acute disseminated encephalomyelitis; TM, transverse myelitis; SPS, stiff-person syndrome; MMN, multifocal motor neuropathy; AE, autoimmune encephalitis; PNS, paraneoplastic syndrome; CDPN, chronic dysimmune peripheral neuropathy; SLE encephalitis, systemic lupus erythematosus encephalitis; NOM, neuromyelitis optica; ON, optic neuritis; MOGAD, myelin oligodendrocyte glycoprotein antibody-associated disease; TS-HDS neuropathy, trisulfated heparan disaccharide IgM antibody-associated peripheral neuropathy; Anti-NMDAR encephalitis, anti-N-methyl-D-aspartate receptor encephalitis; Anti-GABAbR encephalitis, anti-gamma-aminobutyric acid type B receptor encephalitis; Anti-LGI1 encephalitis, anti-leucine-rich glioma-inactivated 1 protein antibody-associated encephalitis; Anti-AMPAR encephalitis, anti-alpha-amino-3-hydroxy-5-methyl-4-isoxazolepropionic acid receptor encephalitis; VGKC-Ab diseases, voltage-gated potassium channel antibody diseases; SLE-E, systemic lupus erythematosus-related encephalitis.

The quality of the studies was assessed using the MINORS scale. The results are presented in the table below. The scores of the included studies ranged from 8 to 14, with the majority around 10, indicating that the overall quality of the included literature was acceptable (Table 2).

**Table 2:** Minors scale scores for each included study.

Study	MINORS scores								Total
	Research with a defined purpose	Patient consistency of inclusion	Anticipated data collection	Outcome indicator response study purpose	Objectivity of outcome indicators	Adequate follow-up time	Lost visit rate below 5%	Estimated sample size	
Aljezani et al. 2024 (39)	2	1	0	2	2	1	2	0	10
Ali et al. 2024 (11)	2	1	0	2	1	1	2	0	9
Błasiak et al. 2025 (12)	2	1	0	2	2	1	2	0	10
Bava et al. 2024 (13)	2	1	1	2	1	2	1	0	10
Carandina-Maffeis et al. 2004 (14)	2	2	0	2	1	2	2	0	11
Chen et al. 2024 (15)	2	2	2	2	1	2	2	0	13
Dogra et al. 2020 (16)	2	1	0	2	1	2	2	0	10
Dada and Kaplan 2004 (17)	2	1	0	2	1	2	2	0	10
Gafoor et al. 2015 (40)	2	1	0	2	1	2	2	0	10
Gala-Błądzińska et al. 2020 (18)	2	1	0	2	1	2	2	0	10
Kaya et al. 2013 (19)	2	1	0	2	1	2	2	0	10
Kaynar et al. 2008 (41)	2	2	0	2	1	2	2	0	11
Korkmaz et al. 2025 (42)	2	1	0	2	1	2	2	0	10
Kumar et al. 2015 (20)	2	2	0	2	1	2	2	0	11
Kumawat et al. 2024 (21)	2	1	0	2	1	2	2	0	10
Lehmann et al. 2008 (22)	2	1	0	2	1	2	2	0	10
Mercure-Corriveau et al. 2023 (44)	2	1	0	2	1	2	2	0	10
Moser et al. 2019 (3)	2	1	0	2	1	2	2	0	10
Naik et al. 2021 (45)	2	1	0	2	1	2	0	0	8
Nieto-Aristizábal et al. 2020 (46)	2	1	0	2	1	2	1	0	9
Pagano et al. 2014 (47)	2	1	0	2	1	2	2	0	10
Seyhanli et al. 2022 (23)	2	1	0	2	1	2	2	0	10
Shah et al. 2020 (24)	2	1	0	2	1	2	2	0	10
Sheckley et al. 2021 (25)	2	1	0	2	1	2	2	0	10
Sinanović et al. 2017 (26)	2	1	0	2	1	2	2	0	10
Tombak et al. 2016 (27)	2	1	0	2	1	2	2	0	10
Yamada et al. 2015 (28)	2	1	0	2	1	2	2	0	10
Yıldırım et al. 2021 (29)	2	1	0	2	1	2	2	0	10
Zhang et al. 2021 (30)	2	2	2	2	2	2	2	0	14
Totzeck et al. 2022 (31)	2	1	0	2	1	2	2	0	10
Fateen et al. 2023 (43)	2	1	0	2	2	2	2	0	11
Olsen et al. 2021 (32)	2	1	0	2	1	2	2	0	10
Codron et al. 2017 (33)	2	1	0	2	1	2	2	0	10
Vikrant et al. 2017 (34)	2	1	0	2	1	2	2	0	10
Pham et al. 2025 (35)	2	1	0	2	1	2	2	0	10
Jamal et al. 2025 (36)	2	1	0	2	2	2	2	0	11
Gupta et al. 2025 (37)	2	2	2	2	1	2	2	0	13
Eichinger et al. 2025 (38)	2	1	0	2	1	2	2	0	10

### 3.3 Clinical response rate

A total of 38 studies were included in this analysis. Among these, 34 studies with 1,722 patients reported clinical response data for overall descriptive pooling; 31 studies with 1,676 patients reported age data for subgroup analysis. Overall heterogeneity was high (*I*^2^ = 76.4%) (Supplementary Table S1), and thus, a random-effects model was used. Due to the high heterogeneity among studies and the definition of clinical response not completely consistent across studies, this pooled result should be interpreted with caution.

#### 3.3.1 Disease subgroup analysis

**Figure 2:**
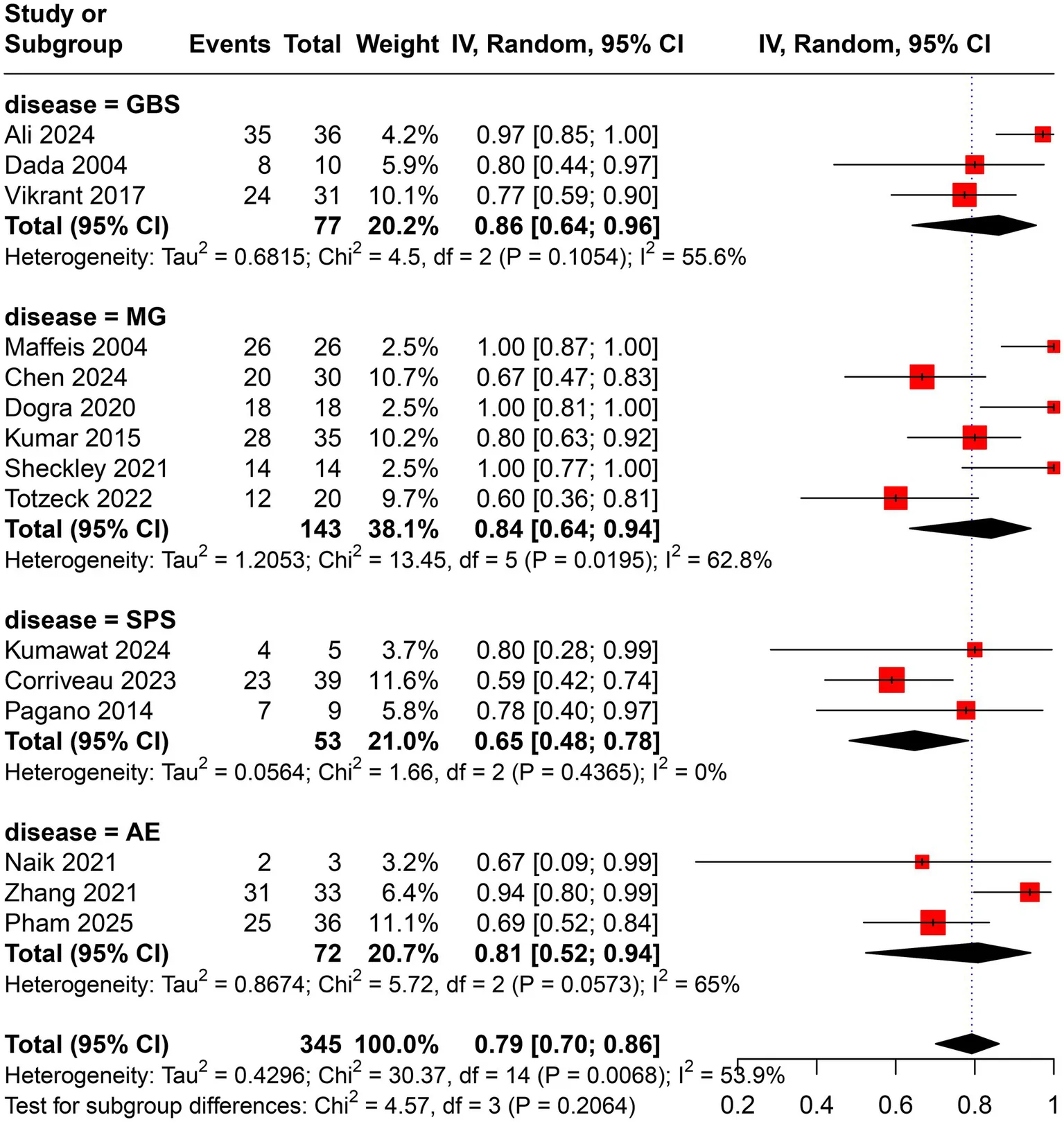
Disease subgroup analysis of response rate.

Response rates were pooled according to subgroups within the same disease. After stratification by disease type, the GBS subgroup had the highest response rate, while the SPS subgroup had a relatively lower response rate. The response rate was 86% (95% CI: 64–96%, *I*^2^ = 55.6%) for the GBS subgroup, 84% (95% CI: 64–94%, *I*^2^ = 62.8%) for the MG subgroup, 81% (95% CI: 52–94%, *I*^2^ = 65%) for the AE subgroup, and 65% (95% CI: 48–78%, *I*^2^ = 0%) for the SPS subgroup. However, the differences among disease subgroups were not statistically significant (*χ*^2^ = 4.57, d*f* = 3, *p* = 0.2064) (Figure 2).

#### 3.3.2 Age subgroup analysis

**Figure 3:**
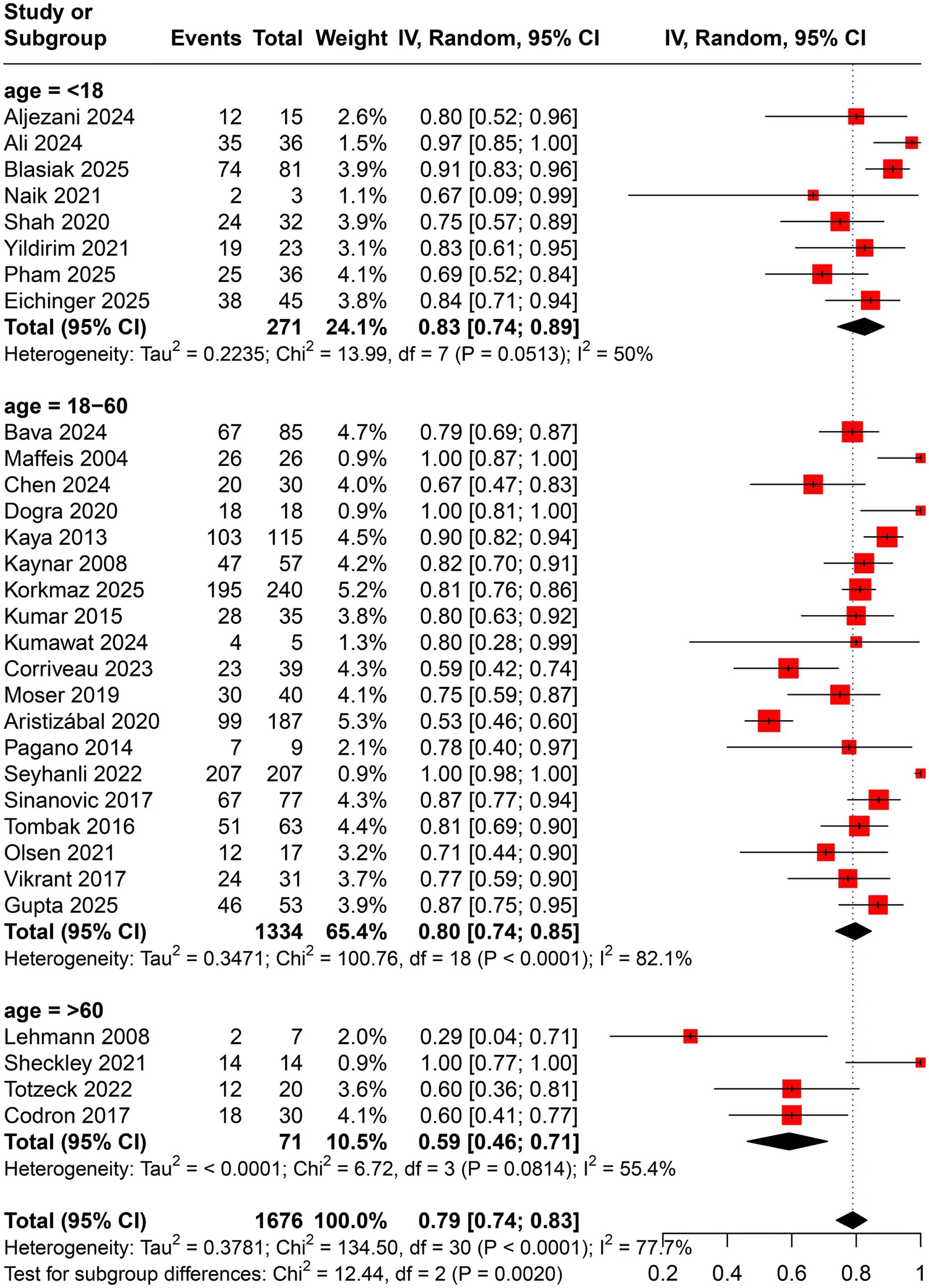
Age subgroup analysis of response rate.

Response rates were pooled according to subgroups defined by the mean or median ages in the original studies. Following stratification by age, the response rate was 83% (95% CI: 74–89%, *I*^2^ = 50.0%) for the <18 years subgroup, 80% (95% CI: 74–85%, *I*^2^ = 82.1%) for the 18–60 years subgroup, and 59% (95% CI: 46–71%, *I*^2^ = 55.4%) for the >60 years subgroup. The variations among age subgroups were statistically significant (*χ*^2^ = 12.44, d*f* = 2, *p* = 0.0020). This suggested that the response rate in the subgroup with the mean or median age >60 years was lower than that in the subgroup with the mean or median age <18 years, possibly related to more comorbid conditions in old patients. However, this analysis was based on study-level or subgroup-level data rather than individual patients’ data. Therefore, the age-related results should still be interpreted with caution (Figure 3).

#### 3.3.3 Region subgroup analysis

**Figure 4:**
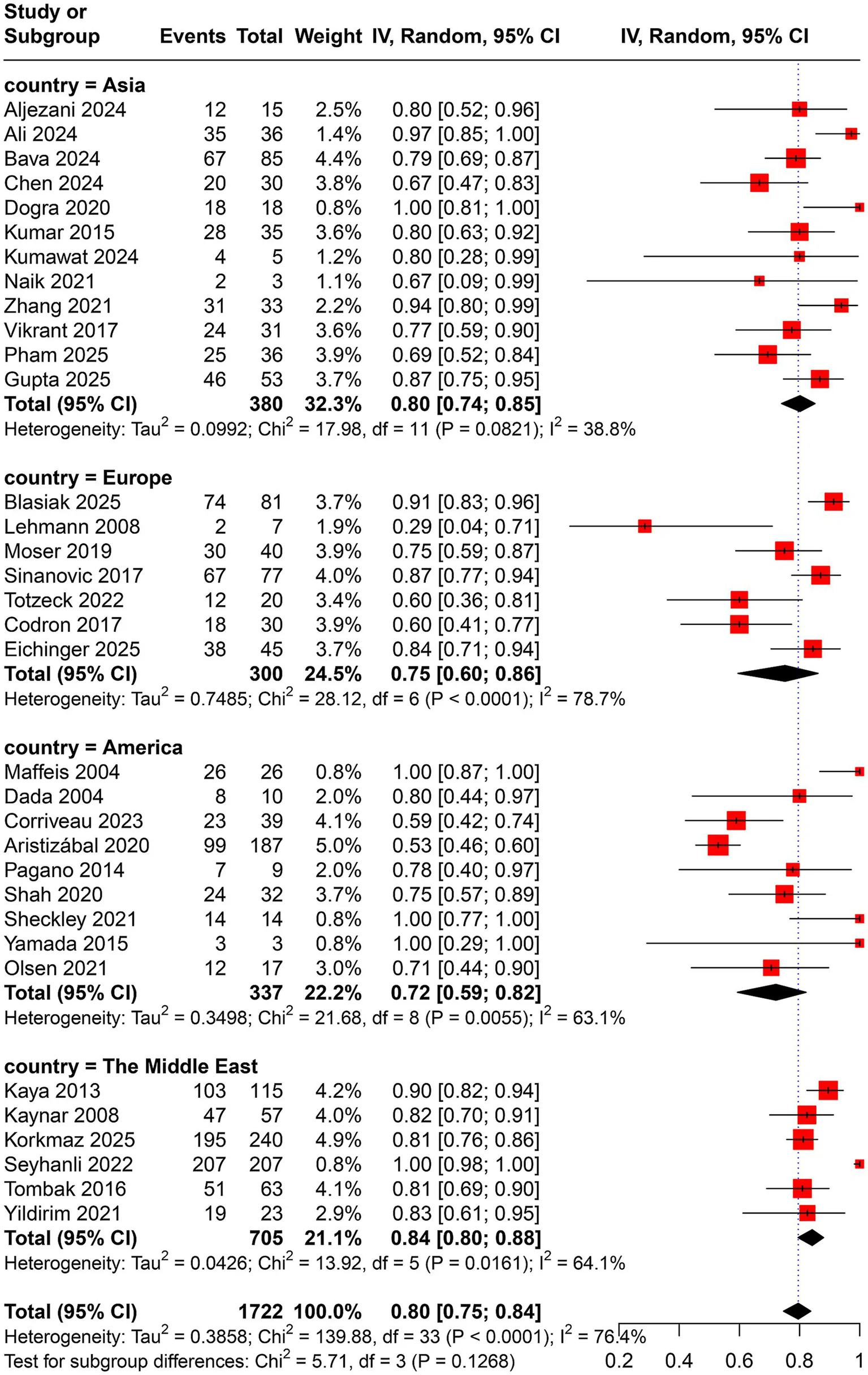
Region subgroup analysis of response rate.

Response rates were pooled according to subgroups defined by geographic regions in the original studies. After stratification by region, the response rate was 84% (95% CI: 80–88%, *I*^2^ = 64.1%) for the Middle East subgroup, 80% (95% CI: 74–85%, *I*^2^ = 38.8%) for the Asia subgroup, 75% (95% CI: 60–86%, *I*^2^ = 78.7%) for the Europe subgroup, and 72% (95% CI: 59–82%, *I*^2^ = 63.1%) for the Americas subgroup. The differences among region subgroups were not statistically significant (*χ*^2^ = 5.71, d*f* = 3, *p* = 0.1268) (Figure 4).

### 3.4 Incidence rate of adverse events

A total of 11 types of adverse events were analyzed in the present study, encompassing fever and chills, dyspnea, allergic reactions, hypocalcemia, arrhythmia, nausea and vomiting, hypertension, hypotension, catheter-related infections, deep vein thrombosis, and coagulation disorders. Some studies documented adverse events as per TPE sessions and others as per the number of patients. The primary results of this study were reported using the incidence rates of adverse events based on the number of patients.

Overall, the pooled incidence rates of various adverse events were not high, but most outcomes showed moderate to high heterogeneity. Hypotension was the most common adverse event (12, 95% CI: 7–19%, *I*^2^ = 93.3%), followed by coagulation disorders (11, 95% CI: 5–23%, *I*^2^ = 80.9%), catheter-related infections (8, 95% CI: 4–15%, *I*^2^ = 93.9%), allergic reactions (7, 95% CI: 4–14%, *I*^2^ = 83.7%), and hypocalcemia (7, 95% CI: 3–16%, *I*^2^ = 93.5%).

The specific pooled results for various adverse events are as follows.

Incidence rates of adverse events based on the number of patients: For fever and chills, the CI was extremely wide, rendering the estimate of little practical value. Dyspnea was reported in 3 studies with 398 patients, with a pooled incidence rate of 3% (95% CI: 0–16%, *I*^2^ = 88.3%). Allergic reactions were documented in 12 studies involving 1,245 patients, with a pooled incidence rate of 7% (95% CI: 4–14%, *I*^2^ = 83.7%). Hypocalcemia was recorded in 10 studies with 861 patients, with a pooled incidence rate of 7% (95% CI: 3–16%, *I*^2^ = 93.5%). Arrhythmia was identified in 6 studies with 711 patients, with a pooled incidence rate of 2% (95% CI: 1–7%, *I*^2^ = 73.8%). Nausea and vomiting were observed in 5 studies involving 606 patients, with a pooled incidence rate of 3% (95% CI: 1–14%, *I*^2^ = 91.0%). Hypertension was noted in 3 studies involving 510 patients, with a pooled incidence rate of 1% (95% CI: 1–3%, *I*^2^ = 0%). Hypotension was reported in 18 studies with 1,733 patients, with a pooled incidence rate of 12% (95% CI: 7–19%, *I*^2^ = 93.3%). Catheter-related infections were recorded in 14 studies involving 1,644 patients, with a pooled incidence rate of 8% (95% CI: 4–15%, *I*^2^ = 93.9%). Deep vein thrombosis was noted in 7 studies with 801 patients, with a pooled incidence rate of 3% (95% CI: 2–6%, *I*^2^ = 50.8%). Coagulation disorders were documented in 8 studies with 621 patients, with a pooled incidence rate of 11% (95% CI: 5–23%, *I*^2^ = 80.9%).

Incidence rates of adverse events based on TPE sessions: Fever and chills were reported in 5 studies with 1,067 exposures, with a pooled incidence rate of 4% (95% CI: 2–10%, *I*^2^ = 86.4%). Dyspnea was recorded in 4 studies involving 1,171 exposures, with a pooled incidence rate of 2% (95% CI: 0–6%, *I*^2^ = 77.8%). Allergic reactions were documented in 7 studies with 1,511 exposures, with a pooled incidence rate of 4% (95% CI: 2–9%, *I*^2^ = 84.9%). Hypocalcemia was observed in 4 studies involving 982 exposures, with a pooled incidence rate of 3% (95% CI: 1–9%, *I*^2^ = 86.5%). Arrhythmia was identified in 5 studies with 946 exposures, with a pooled incidence rate of 3% (95% CI: 1–13%, *I*^2^ = 93.2%). Nausea and vomiting were noted in 3 studies involving 1,049 exposures, with a pooled incidence rate of 2% (95% CI: 0–9%, *I*^2^ = 84.0%). Hypertension was reported in 3 studies with 602 exposures, with a pooled incidence rate of 3% (95% CI: 1–6%, *I*^2^ = 45.2%). Hypotension was recorded in 9 studies with 1,935 exposures, with a pooled incidence rate of 8% (95% CI: 3–18%, *I*^2^ = 93.3%). Catheter-related infections were documented in 6 studies involving 1,248 exposures, with a pooled incidence rate of 3% (95% CI: 2–5%, *I*^2^ = 21.5%). Deep vein thrombosis was not reported. For coagulation disorders, the CI was extremely wide, rendering the estimate of little practical value. The summary of all adverse events is presented in Table 3.

**Table 3:** Overall incidence rates of adverse events.

Outcomes	Incidence rates of adverse events based on the number of patients				Incidence rates of adverse events based on TPE sessions			
	Number of included studies/*n*	Effect size	95% CI	Heterogeneity	Number of included studies/*n*	Effect size	95% CI	Heterogeneity
Fever and chills	CI extremely wide, of little practical value.				5	4%	2–10%	84.5%
Dyspnea	3	3%	0–16%	88.3%	4	2%	0–6%	77.8%
Allergic reactions	12	7%	4–14%	83.7%	7	4%	2–9%	84.9%
Hypocalcemia	10	7%	3–16%	93.5%	4	3%	1–9%	86.5%
Arrhythmia	6	2%	1–7%	73.8%	5	3%	1–13%	93.2%
Nausea and vomiting	5	3%	1–14%	91.0%	3	2%	0–9%	84.0%
Hypertension	3	1%	1–3%	0%	3	3%	1–6%	45.2%
Hypotension	18	12%	7–19%	93.3%	9	8%	3–18%	93.3%
Catheter-related infections	14	8%	4–15%	93.9%	6	3%	2–5%	21.5%
Deep vein thrombosis	7	3%	2–6%	50.8%	—	—	—	—
Coagulation disorders	8	11%	5–23%	80.9%	CI extremely wide, of little practical value.			

### 3.5 Mortality assessment

In total, 2,199 individuals were incorporated in the present study, with 49 deaths reported. Pooled analysis showed that the all-cause in-hospital mortality rate among patients undergoing TPE was 4% (95% CI: 3–5%), with between-study heterogeneity of *I*^2^ = 36.5%, and this value does not represent mortality attributable to TPE. According to the original studies, most deaths were linked to the progression of the primary disease or related complications and were not definitively attributed to the TPE procedure itself. Therefore, this result reflected the overall death status in individuals receiving TPE and should not be directly interpreted as the TPE-related mortality rate (Supplementary Figure S1).

### 3.6 Sensitivity analysis and publication bias

#### 3.6.1 Overall response rate across heterogeneous individual studies

Due to significant heterogeneity in the criteria for defining clinical effectiveness across all included studies, the pooled response rate for all diseases calculated in this study is presented only for descriptive purposes. The leave-one-out sensitivity analysis uncovered that after the removal of any single study, the response rate remained stable at 79–81%, and *I*^2^ fluctuated between 59.8 and 76.7%. This indicated that the overall results were relatively robust. In the publication bias analysis, the funnel plot and Egger’s test suggested potential publication bias (*p* = 0.0076). After further adjustment using the trim-and-fill method, the pooled response rate did not change substantially. Thus, the impact of potential publication bias on the overall results might be limited (Supplementary Figure S2).

#### 3.6.2 Mortality rate

The leave-one-out sensitivity analysis for mortality rates demonstrated that after the elimination of any individual study, the pooled mortality rate changed only slightly, and I^2^ fluctuated between 20.8–38.2%. This revealed that the overall results were relatively stable. In the publication bias analysis, the funnel plot and Egger’s test indicated potential publication bias (*p* = 0.0039). After adjustment utilizing the trim-and-fill method, the pooled mortality rate did not vary considerably. Consequently, the influence of potential publication bias on the mortality results might be limited (Supplementary Figure S3).

## 4 Discussion

The present study systematically integrated published real-world observational studies and eligible single-arm clinical trials to evaluate the clinical response rate, mortality rate, and incidence of adverse events associated with TPE in the treatment of ANDs. The results showed that the clinical response rates were relatively high across subgroups after TPE. This study is an exploratory descriptive analysis based on heterogeneous definitions. The pooled response rate is only shown in Supplementary Figure S4 and should not be regarded as an estimate derived from a unified response criterion. and hypotension was the most commonly reported adverse event. Given that the included studies are predominantly single-arm or retrospective in design, and due to variations in disease types, baseline patient characteristics, treatment protocols, and definition of efficacy, the findings should be interpreted as a comprehensive description of the existing evidence and do not allow direct inferences regarding the comparative efficacy of TPE relative to other treatment modalities.

This study identified the pooled response rate in disease subgroups as the primary outcome. The response rates were 86% for GBS, 84% for MG, 81% for AE, and 65% for SPS. This finding suggests that, in real-world and single-arm studies, individuals with ANDs treated with TPE show a relatively high rate of clinical improvement. This result is generally consistent with the findings of Tombak et al. and Korkmaz et al., which demonstrate varying degrees of clinical improvement in individuals with various neuroimmunological diseases following TPE (27, 42). Disease subgroup analysis uncovered that the GBS subgroup had a numerically higher clinical response rate, while the SPS subgroup had a relatively lower response rate. However, the variations between disease subgroups were not statistically significant (*p* = 0.2064). Previous studies have also indicated that GBS is one of the more common indications for TPE with a relatively robust evidence base (40, 41). A possible reason for the good response to TPE in individuals with GBS is that its pathological process is often related to pathogenic antibodies, complement components, and inflammatory mediators in the circulation, and TPE can rapidly remove these plasma components, thereby alleviating immune-mediated nerve injury (48). For individuals with MG, TPE can clear pathogenic antibodies like acetylcholine receptor antibodies from the circulation (49) and thus has a certain clinical value in crisis or acute exacerbations of MG. Nevertheless, the differences between disease subgroups in the present study was not statistically significant, and most of the included studies were single-arm in design. Therefore, it cannot be concluded that one disease subtype is necessarily superior to another in terms of efficacy based on these findings.

Age subgroup analysis showed that the pooled response rate in the subgroups with mean or median ages <18 years was higher than that in the subgroups with mean or median ages >60 years, and the variation between subgroups was statistically significant (*p* = 0.0020). This finding reflects that age group may be linked to clinical response after TPE. However, because this study was an analysis at the study level or subgroup level rather than at the level of individual patients’ data, it does not prove that age itself is an independent factor influencing the efficacy of TPE. The higher response rate in subgroups with mean or median ages <18 years may be related to factors like composition of the disease spectrum, earlier initiation of treatment, fewer underlying comorbidities, lower burden of chronic neurological damage, and better potential for neurological recovery (50). In contrast, old patients may have more underlying diseases, higher infection risk, cardiovascular instability, or more severe baseline neurological damage, and all these factors may affect clinical recovery. Thus, the age subgroup findings are more appropriately considered as hypothesis-generating observations and should be further validated in the future based on individual patients’ data or prospective cohort studies.

Region subgroup analysis demonstrated certain numerical differences in response rates across distinct regions, and a relatively higher response rate was identified in the Middle East. However, the differences between region subgroups were not statistically significant (*p* = 0.1268). This finding suggests that the efficacy of TPE in different regions may be influenced by variations in disease spectrum, referral patterns, timing of treatment, healthcare resources, operational protocols, and follow-up management. Nevertheless, existing evidence is insufficient to support clear regional differences. Therefore, the region subgroup results should be regarded as trend observations reflecting real-world clinical heterogeneity rather than being interpreted as evidence of superior TPE efficacy in any particular region.

A total of 49 deaths were reported in this study. Pooled analysis showed that the all-cause in-hospital mortality rate among patients undergoing TPE was 4% (95% CI: 3–5%, *I*^2^ = 36.5%), and this value does not represent mortality attributable to TPE. According to the original studies, most deaths were related to disease progression, respiratory failure, infection, or other serious complications, and were not explicitly attributed to the TPE procedure itself. Thus, the mortality result reported in the present study should be interpreted as an indicator reflecting the overall death status among individuals receiving TPE, rather than the TPE-related mortality rate. Due to the general lack of standardized assessment of causes of death in the included studies, this study cannot accurately evaluate the independent effect of the TPE procedure itself on mortality risk.

In terms of safety, the present study found that adverse events related to TPE or reported during treatment were predominantly mild to moderate, including hypotension, allergic reactions, catheter-related infections, hypocalcemia, and coagulation disorders. Among these, hypotension was the most common adverse event, with a pooled incidence rate of 12%. This finding is consistent with results from previous studies, which suggest that hypotension is one of the more common complications during TPE (43). TPE-related adverse events may be associated with vascular access, blood volume changes, replacement fluid type, anticoagulation regimen, and procedural process (39, 51). For example, citrate anticoagulation may lead to hypocalcemia, blood volume fluctuations can induce hypotension, and the use of central venous catheters may increase the risk of infection or thrombosis. With standardized procedures and close monitoring, most adverse events can be promptly identified and managed. However, this does not mean that TPE is entirely without risk.

In the present study, most adverse event outcomes showed moderate to high heterogeneity, which may be attributed to the following factors. First, the included studies were predominantly real-world observational studies and single-arm trials, leading to inconsistencies in patient selection, treatment indications, and procedural protocols. Second, there was a substantial variation in disease subtypes, and individuals with different diseases differed in levels of disease severity, immune activity status, and risk of complications. Third, TPE treatment protocols differed across studies, including the number of sessions, exchange volume, replacement fluid type, anticoagulation method, vascular access, and combined treatment regimens. Fourth, the definitions of clinical response and adverse events, as well as the intensity of monitoring and methods of reporting, varied among studies. Fifth, differences in healthcare resources, level of care, and experience with TPE procedures may exist across geographic regions. In this study, heterogeneity was examined through subgroup analyses by disease, age, region, and statistical approach of adverse events, and the robustness of the results was appraised using leave-one-out sensitivity analysis.

This study has several strengths. First, it systematically integrated real-world evidence from individuals with various ANDs treated with TPE, covering different disease types, age groups, and geographic regions, which helps reflect the application of TPE in clinical practice. Second, the present study simultaneously evaluated the response rate in disease subgroups, mortality rates, and multiple adverse events, providing a relatively comprehensive profile of the efficacy and safety of TPE. Third, subgroup analyses by disease type, age, region, and statistical approach of adverse events, as well as sensitivity analysis and publication bias assessment, enhance the interpretative robustness of the findings. However, because most of the included studies were single-arm or retrospective in design, these strengths cannot replace the causal inference evidence provided by high-quality controlled studies.

The present study also has several limitations. First, most of the included studies were retrospective or single-arm in design, lacking a control group. This precludes direct comparisons of the relative efficacy and safety of TPE and IVIG, corticosteroids, immuno-adsorption, or other treatment strategies. Second, the definition of clinical response varied across studies, and some studies used disease-specific criteria, symptom relief, or physician judgment as measures of efficacy, which may affect the comparability of the pooled response rate. Third, reporting methods for adverse events were inconsistent. Some studies reported adverse events as per the number of patients, while others reported such events as per TPE sessions. Fourth, TPE was often used in combination with corticosteroids, IVIG, immuno-suppressants, or intensive supportive care, and this study could not distinguish the independent contribution of TPE itself and that of combined therapies to clinical improvement. Fifth, there were differences across studies in the timing of TPE initiation, number of sessions, exchange volume, replacement fluid type, anticoagulation method, and choice of vascular access. However, due to insufficient data, adequate subgroup analyses based on these procedural parameters could not be performed. Sixth, publication bias analysis suggested potential publication bias for the primary outcome and mortality rates. Although the pooled results did not change substantially after adjustment by the trim-and-fill method, the findings should be interpreted with caution. Finally, the MINORS scores indicated that most included studies were moderate in methodological quality, which may lower the certainty of the evidence. Thus, it should not be assumed that the moderate quality has no impact on the reliability of the primary outcome.

## 5 Conclusion

In conclusion, existing real-world observational studies and eligible single-arm clinical trials indicate that patients with ANDs show a relatively high rate of clinical improvement after TPE. The reported adverse events are predominantly manageable events such as hypotension, suggesting acceptable overall safety. The deaths reported in the included studies are mostly related to the progression of the primary disease or related complications rather than being explicitly attributed to the TPE procedure itself. Given that most of the included studies are single-arm or retrospective in design and inconsistencies exist in efficacy definitions and safety reporting across studies, more high-quality, multicenter studies are needed in the future to further define the appropriate patient population, optimal treatment regimen, and long-term safety of TPE.

## Data Availability

The original contributions presented in the study are included in the article/Supplementary material, further inquiries can be directed to the corresponding authors.

## Glossary

ANDs: autoimmune neurological diseases
GBS: Guillain-Barre syndrome
MG: myasthenia gravis
MS: multiple sclerosis
NMOSD: neuromyelitis optica spectrum disorder
CIDP: chronic inflammatory demyelinating polyneuropathy
AIDP: acute inflammatory demyelinating polyneuropathy (subtype of GBS)
ADEM: acute disseminated encephalomyelitis
TM: transverse myelitis
SPS: stiff-person syndrome
MMN: multifocal motor neuropathy
AE: autoimmune encephalitis
PNS: paraneoplastic syndrome
CDPN: chronic dysimmune peripheral neuropathy
SLE encephalitis: systemic lupus erythematosus encephalitis
NOM: neuromyelitis optica
ON: optic neuritis
MOGAD: myelin oligodendrocyte glycoprotein antibody-associated disease
TS-HDS neuropathy: trisulfated heparan disaccharide IgM antibody-associated peripheral neuropathy
Anti-NMDAR encephalitis: anti-N-methyl-D-aspartate receptor encephalitis
Anti-GABAbR encephalitis: anti-gamma-aminobutyric acid type B receptor encephalitis
Anti-LGI1 encephalitis: anti-leucine-rich glioma-inactivated 1 protein antibody-associated encephalitis
Anti-AMPAR encephalitis: anti-alpha-amino-3-hydroxy-5-methyl-4-isoxazolepropionic acid receptor encephalitis
VGKC-Ab diseases: voltage-gated potassium channel antibody diseases
SLE-E: systemic lupus erythematosus-related encephalitis
FFP: fresh frozen plasma
HA: human albumin
IVIG: intravenous immunoglobulin
HES: hydroxyethyl starch
SG: succinylated gelatin
