# Supplementary material for: Effects of therapeutic plasma exchange (TPE) on efficacy and adverse events in patients with autoimmune neurological diseases: a systematic review and meta-analysis based on real-world studies and single-arm clinical trials
Source: Front Neurol. 2026 Aug 26;17:1916922. doi: 10.3389/fneur.2026.1916922 (PMC13553445; doi:10.3389/fneur.2026.1916922)
Supplement: Supplementary file 1 [file Supplementary_file_1.docx]

**Supplementary materials**

**Table S1.** Search strategy for PubMed, Embase, Cochrane

| Database | Search detail | Results | Time |
| --- | --- | --- | --- |
| Pubmed | ((((((((((((((((((((((((((((((((((((Myasthenia Gravis[Title/Abstract]) OR (Guillain-Barre Syndrome[Title/Abstract])) OR (Acute inflammatory demyelinating polyradiculoneuropathy[Title/Abstract])) OR (Miller Fisher Syndrome[Title/Abstract])) OR (Chronic Acquired Demyelinating Polyneuropathies[Title/Abstract])) OR (Chronic Inflammatory Demyelinating Polyradiculoneuropathy[Title/Abstract])) OR (Polyneuropathy[Title/Abstract])) OR (Demyelinating encephalopathy[Title/Abstract])) OR (demyelinating neuropathy[Title/Abstract])) OR (Stiff-Person Syndrome[Title/Abstract])) OR (Moersch Woltmann[Title/Abstract])) OR (Polyradiculoneuropathy[Title/Abstract])) OR (polyneuritis[Title/Abstract])) OR (Sensory Neuropathy[Title/Abstract])) OR (Peripheral Neuropathy[Title/Abstract])) OR (Motor Neuropathy[Title/Abstract])) OR (Sensorimotor Neuropathy[Title/Abstract])) OR (Lambert-Eaton Myasthenic Syndrome[Title/Abstract])) OR (Paraneoplastic Neurologic Syndromes[Title/Abstract])) OR (Paraneoplastic Neuropathy[Title/Abstract])) OR (paraneoplastic cerebellar degeneration[Title/Abstract])) OR (paraneoplastic encephalitis[Title/Abstract])) OR (limbic encephalitis[Title/Abstract])) OR (opsoclonus myoclonus[Title/Abstract])) OR (paraneoplastic opsoclonus[Title/Abstract])) OR (Autoimmune Encephalitis[Title/Abstract])) OR (Autoimmune Thyroiditis[Title/Abstract])) OR (Anti NMDA Receptor Encephalitis[Title/Abstract])) OR (Anti-N-Methyl-D-Aspartate Receptor Encephalitis[Title/Abstract])) OR (Multifocal motor neuropathy[Title/Abstract])) OR (Multifocal acquired demyelinating sensory[Title/Abstract] AND motor neuropathy[Title/Abstract])) OR (Neuropathy associated monoclonal IgM antibodies myelin-associated glycoprotein[Title/Abstract])) OR (Myelin-Associated Glycoprotein neuropathy[Title/Abstract] AND Hashimoto’s encephalopathy[Title/Abstract])) OR (steroid-responsive encephalopathy associated with autoimmune thyroiditis[Title/Abstract])) OR (autoimmune neurological diseases[Title/Abstract])) AND ((((Plasma Exchange[Title/Abstract]) OR (Exchange, Plasma[Title/Abstract])) OR (Exchanges, Plasma[Title/Abstract])) OR (Plasma Exchanges[Title/Abstract]))) AND ((((retrospective[Title/Abstract]) OR (observational[Title/Abstract])) OR (prospective[Title/Abstract])) OR (real world[Title/Abstract])) | 253 | 2025.03.03 |
| Embase | #1 'myasthenia gravis':ti,ab,kw OR 'guillain-barre syndrome':ti,ab,kw OR 'acute inflammatory demyelinating polyradiculoneuropathy':ti,ab,kw OR 'miller fisher syndrome':ti,ab,kw OR 'chronic acquired demyelinating polyneuropathies':ti,ab,kw OR 'chronic inflammatory demyelinating polyradiculoneuropathy':ti,ab,kw OR polyneuropathy:ti,ab,kw OR 'demyelinating encephalopathy':ti,ab,kw OR 'demyelinating neuropathy':ti,ab,kw OR 'stiff-person syndrome':ti,ab,kw OR 'moersch woltmann':ti,ab,kw OR polyradiculoneuropathy:ti,ab,kw OR polyneuritis:ti,ab,kw OR 'sensory neuropathy':ti,ab,kw OR 'peripheral neuropathy':ti,ab,kw OR 'motor neuropathy':ti,ab,kw OR 'sensorimotor neuropathy':ti,ab,kw OR 'lambert-eaton myasthenic syndrome':ti,ab,kw OR 'paraneoplastic neurologic syndromes':ti,ab,kw OR 'paraneoplastic neuropathy':ti,ab,kw OR 'paraneoplastic cerebellar degeneration':ti,ab,kw OR 'paraneoplastic encephalitis':ti,ab,kw OR 'limbic encephalitis':ti,ab,kw OR 'opsoclonus myoclonus':ti,ab,kw OR 'paraneoplastic opsoclonus':ti,ab,kw OR 'autoimmune encephalitis':ti,ab,kw OR 'autoimmune thyroiditis':ti,ab,kw OR 'anti nmda receptor encephalitis':ti,ab,kw OR 'anti-n-methyl-d-aspartate receptor encephalitis':ti,ab,kw OR 'multifocal motor neuropathy':ti,ab,kw OR 'multifocal acquired demyelinating sensory':ti,ab,kw OR 'neuropathy associated with monoclonal igm antibodies to myelin-associated glycoprotein':ti,ab,kw OR 'myelin-associated glycoprotein neuropathy':ti,ab,kw OR 'steroid-responsive encephalopathy associated with autoimmune thyroiditis':ti,ab,kw OR 'autoimmune neurological diseases':ti,ab,kw #2 'plasma exchange':ti,ab,kw OR 'exchange, plasma':ti,ab,kw OR 'exchanges, plasma':ti,ab,kw OR 'plasma exchanges':ti,ab,kw #3 retrospective:ti,ab,kw OR observational:ti,ab,kw OR 'real world':ti,ab,kw #4 #1 AND #2 AND #3 | 464 | 2025.03.03 |
| Cochrane | #1 (Myasthenia Gravis);ti,ab,kw OR (Guillain-Barre Syndrome):ti,ab,kw OR (Acute inflammatory demyelinating polyradiculoneuropathy):ti,ab,kw OR (Miller Fisher Syndrome):ti,ab,kw OR (Chronic Acquired Demyelinating Polyneuropathies):ti,ab,kw #2 (Chronic Inflammatory Demyelinating Polyradiculoneuropathy):ti,ab,kw OR (Polyneuropathy):ti,ab,kw OR (Demyelinating encephalopathy):ti,ab,kw OR (demyelinating neuropathy):ti,ab,kw OR (Stiff-Person Syndrome):ti,ab,kw #3 (Moersch Woltmann):ti,ab,kw OR (Polyradiculoneuropathy):ti,ab,kw OR (polyneuritis):ti,ab,kw OR (Sensory Neuropathy):ti,ab,kw OR (Peripheral Neuropathy):ti,ab,kw #4 (Motor Neuropathy):ti,ab,kw OR (Sensorimotor Neuropathy):ti,ab,kw OR (Lambert-Eaton Myasthenic Syndrome):ti,ab,kw OR (Paraneoplastic Neurologic Syndromes):ti,ab,kw OR (Paraneoplastic Neuropathy):ti,ab,kw #5 (paraneoplastic cerebellar degeneration):ti,ab,kw OR (paraneoplastic encephalitis):ti,ab,kw OR (limbic encephalitis):ti,ab,kw OR (opsoclonus myoclonus):ti,ab,kw OR (paraneoplastic opsoclonus):ti,ab,kw #6 (Autoimmune Encephalitis):ti,ab,kw OR (Autoimmune Thyroiditis):ti,ab,kw OR (Anti NMDA Receptor Encephalitis):ti,ab,kw OR (Anti-N-Methyl-D-Aspartate Receptor Encephalitis):ti,ab,kw OR (Multifocal motor neuropathy):ti,ab,kw #7 (Multifocal acquired demyelinating sensory and motor neuropathy):ti,ab,kw OR (Neuropathy associated monoclonal IgM antibodies myelin-associated glycoprotein):ti,ab,kw OR (Myelin-Associated Glycoprotein neuropathy and Hashimoto’s encephalopathy):ti,ab,kw OR (steroid-responsive encephalopathy associated with autoimmune thyroiditis):ti,ab,kw OR (autoimmune neurological diseases):ti,ab,kw #8 #1 OR #2 OR #3 OR #4 OR #5 OR #6 OR #7 #9 (Plasma Exchange):ti,ab,kw OR (Exchange, Plasma):ti,ab,kw OR (Exchanges, Plasma):ti,ab,kw OR (Plasma Exchanges):ti,ab,kw #10 (retrospective):ti,ab,kw OR (observational):ti,ab,kw OR (prospective):ti,ab,kw OR (real world):ti,ab,kw #11 #8 AND #9 AND #10 | 105 | 2025.03.03 |
| Pubmed | ((((((((((((((((((((((((((((((((((((Myasthenia Gravis[Title/Abstract]) OR (Guillain-Barre Syndrome[Title/Abstract])) OR (Acute inflammatory demyelinating polyradiculoneuropathy[Title/Abstract])) OR (Miller Fisher Syndrome[Title/Abstract])) OR (Chronic Acquired Demyelinating Polyneuropathies[Title/Abstract])) OR (Chronic Inflammatory Demyelinating Polyradiculoneuropathy[Title/Abstract])) OR (Polyneuropathy[Title/Abstract])) OR (Demyelinating encephalopathy[Title/Abstract])) OR (demyelinating neuropathy[Title/Abstract])) OR (Stiff-Person Syndrome[Title/Abstract])) OR (Moersch Woltmann[Title/Abstract])) OR (Polyradiculoneuropathy[Title/Abstract])) OR (polyneuritis[Title/Abstract])) OR (Sensory Neuropathy[Title/Abstract])) OR (Peripheral Neuropathy[Title/Abstract])) OR (Motor Neuropathy[Title/Abstract])) OR (Sensorimotor Neuropathy[Title/Abstract])) OR (Lambert-Eaton Myasthenic Syndrome[Title/Abstract])) OR (Paraneoplastic Neurologic Syndromes[Title/Abstract])) OR (Paraneoplastic Neuropathy[Title/Abstract])) OR (paraneoplastic cerebellar degeneration[Title/Abstract])) OR (paraneoplastic encephalitis[Title/Abstract])) OR (limbic encephalitis[Title/Abstract])) OR (opsoclonus myoclonus[Title/Abstract])) OR (paraneoplastic opsoclonus[Title/Abstract])) OR (Autoimmune Encephalitis[Title/Abstract])) OR (Autoimmune Thyroiditis[Title/Abstract])) OR (Anti NMDA Receptor Encephalitis[Title/Abstract])) OR (Anti-N-Methyl-D-Aspartate Receptor Encephalitis[Title/Abstract])) OR (Multifocal motor neuropathy[Title/Abstract])) OR (Multifocal acquired demyelinating sensory[Title/Abstract] AND motor neuropathy[Title/Abstract])) OR (Neuropathy associated monoclonal IgM antibodies myelin-associated glycoprotein[Title/Abstract])) OR (Myelin-Associated Glycoprotein neuropathy[Title/Abstract] AND Hashimoto’s encephalopathy[Title/Abstract])) OR (steroid-responsive encephalopathy associated with autoimmune thyroiditis[Title/Abstract])) OR (autoimmune neurological diseases[Title/Abstract])) AND ((((Plasma Exchange[Title/Abstract]) OR (Exchange, Plasma[Title/Abstract])) OR (Exchanges, Plasma[Title/Abstract])) OR (Plasma Exchanges[Title/Abstract]))) AND ((((retrospective[Title/Abstract]) OR (observational[Title/Abstract])) OR (prospective[Title/Abstract])) OR (real world[Title/Abstract])) | 266 | 2026.01.06 |
| Embase | #1 'myasthenia gravis':ti,ab,kw OR 'guillain-barre syndrome':ti,ab,kw OR 'acute inflammatory demyelinating polyradiculoneuropathy':ti,ab,kw OR 'miller fisher syndrome':ti,ab,kw OR 'chronic acquired demyelinating polyneuropathies':ti,ab,kw OR 'chronic inflammatory demyelinating polyradiculoneuropathy':ti,ab,kw OR polyneuropathy:ti,ab,kw OR 'demyelinating encephalopathy':ti,ab,kw OR 'demyelinating neuropathy':ti,ab,kw OR 'stiff-person syndrome':ti,ab,kw OR 'moersch woltmann':ti,ab,kw OR polyradiculoneuropathy:ti,ab,kw OR polyneuritis:ti,ab,kw OR 'sensory neuropathy':ti,ab,kw OR 'peripheral neuropathy':ti,ab,kw OR 'motor neuropathy':ti,ab,kw OR 'sensorimotor neuropathy':ti,ab,kw OR 'lambert-eaton myasthenic syndrome':ti,ab,kw OR 'paraneoplastic neurologic syndromes':ti,ab,kw OR 'paraneoplastic neuropathy':ti,ab,kw OR 'paraneoplastic cerebellar degeneration':ti,ab,kw OR 'paraneoplastic encephalitis':ti,ab,kw OR 'limbic encephalitis':ti,ab,kw OR 'opsoclonus myoclonus':ti,ab,kw OR 'paraneoplastic opsoclonus':ti,ab,kw OR 'autoimmune encephalitis':ti,ab,kw OR 'autoimmune thyroiditis':ti,ab,kw OR 'anti nmda receptor encephalitis':ti,ab,kw OR 'anti-n-methyl-d-aspartate receptor encephalitis':ti,ab,kw OR 'multifocal motor neuropathy':ti,ab,kw OR 'multifocal acquired demyelinating sensory':ti,ab,kw OR 'neuropathy associated with monoclonal igm antibodies to myelin-associated glycoprotein':ti,ab,kw OR 'myelin-associated glycoprotein neuropathy':ti,ab,kw OR 'steroid-responsive encephalopathy associated with autoimmune thyroiditis':ti,ab,kw OR 'autoimmune neurological diseases':ti,ab,kw #2 'plasma exchange':ti,ab,kw OR 'exchange, plasma':ti,ab,kw OR 'exchanges, plasma':ti,ab,kw OR 'plasma exchanges':ti,ab,kw #3 retrospective:ti,ab,kw OR observational:ti,ab,kw OR 'real world':ti,ab,kw #4 #1 AND #2 AND #3 | 472 | 2026.01.06 |
| Cochrane | #1 (Myasthenia Gravis);ti,ab,kw OR (Guillain-Barre Syndrome):ti,ab,kw OR (Acute inflammatory demyelinating polyradiculoneuropathy):ti,ab,kw OR (Miller Fisher Syndrome):ti,ab,kw OR (Chronic Acquired Demyelinating Polyneuropathies):ti,ab,kw #2 (Chronic Inflammatory Demyelinating Polyradiculoneuropathy):ti,ab,kw OR (Polyneuropathy):ti,ab,kw OR (Demyelinating encephalopathy):ti,ab,kw OR (demyelinating neuropathy):ti,ab,kw OR (Stiff-Person Syndrome):ti,ab,kw #3 (Moersch Woltmann):ti,ab,kw OR (Polyradiculoneuropathy):ti,ab,kw OR (polyneuritis):ti,ab,kw OR (Sensory Neuropathy):ti,ab,kw OR (Peripheral Neuropathy):ti,ab,kw #4 (Motor Neuropathy):ti,ab,kw OR (Sensorimotor Neuropathy):ti,ab,kw OR (Lambert-Eaton Myasthenic Syndrome):ti,ab,kw OR (Paraneoplastic Neurologic Syndromes):ti,ab,kw OR (Paraneoplastic Neuropathy):ti,ab,kw #5 (paraneoplastic cerebellar degeneration):ti,ab,kw OR (paraneoplastic encephalitis):ti,ab,kw OR (limbic encephalitis):ti,ab,kw OR (opsoclonus myoclonus):ti,ab,kw OR (paraneoplastic opsoclonus):ti,ab,kw #6 (Autoimmune Encephalitis):ti,ab,kw OR (Autoimmune Thyroiditis):ti,ab,kw OR (Anti NMDA Receptor Encephalitis):ti,ab,kw OR (Anti-N-Methyl-D-Aspartate Receptor Encephalitis):ti,ab,kw OR (Multifocal motor neuropathy):ti,ab,kw #7 (Multifocal acquired demyelinating sensory and motor neuropathy):ti,ab,kw OR (Neuropathy associated monoclonal IgM antibodies myelin-associated glycoprotein):ti,ab,kw OR (Myelin-Associated Glycoprotein neuropathy and Hashimoto’s encephalopathy):ti,ab,kw OR (steroid-responsive encephalopathy associated with autoimmune thyroiditis):ti,ab,kw OR (autoimmune neurological diseases):ti,ab,kw #8 #1 OR #2 OR #3 OR #4 OR #5 OR #6 OR #7 #9 (Plasma Exchange):ti,ab,kw OR (Exchange, Plasma):ti,ab,kw OR (Exchanges, Plasma):ti,ab,kw OR (Plasma Exchanges):ti,ab,kw #10 (retrospective):ti,ab,kw OR (observational):ti,ab,kw OR (prospective):ti,ab,kw OR (real world):ti,ab,kw #11 #8 AND #9 AND #10 | 118 | 2026.01.06 |

**Table S2.** Efficacy Evaluation Indicators and Definition of Treatment Response

| **Study** | **Efficacy evaluation indicators** | **Definition of response** | **Disease type** |
| --- | --- | --- | --- |
| **Aljezani 2024** | Glasgow Coma Scale (GCS) | An increase in GCS after treatment indicates improvement in consciousness. | Various neuroimmunological diseases |
|  | Expanded Disability Status Scale (EDSS) | A decrease in EDSS after treatment indicates relief of neurological disabilities, including limb functions, swallowing, and motor skills. |  |
| **Ali 2024** | MRC muscle strength grading | At least a 1-grade improvement in muscle strength in all children is used as the core criterion for treatment response. | GBS: 36 |
|  | Alleviation of clinical symptoms |  |  |
| **Błasiak 2025** | Hughes Functional Grading Scale (HFGS) | It is applicable to children with GB/PN/MG, with grades ranging from 0 (healthy) to 6 (death). Lower scores indicate better neurological functions. Improvements are assessed by comparing HFGS scores before and after TPE. | Various neuroimmunological diseases |
|  | Author‑developed scale (AS) |  |  |
|  |  | AS: The scoring system is set according to disease groups, with a total score range of –7 to 15. Higher scores indicate milder disease. |  |
| **Bava 2024** | Modified Rankin Scale (mRS) | A decrease in mRS score after treatment. | Various neuroimmunological diseases |
| **Maffeis 2004** | Osserman & Genkins (O&G) classification | Immediate improvement in muscle strength and immediate alleviation of bulbar palsy and respiratory symptoms after a single exchange. | MG: 26 |
|  | Clinical classification of the Myasthenia Gravis Foundation of America (MGFA) |  |  |
| **Chen 2024** | Quantitative Myasthenia Gravis score (QMGs) and/or Myasthenia Gravis Composite score (MGCs) | A decrease of ≥5 points in either score or a reduction of ≥30% from the baseline after treatment is defined as an effective response; a decrease <5 points/<30% or worsening symptoms is defined as a non‑response. | MG: 30 |
| **Dogra 2020** | MGFA clinical classification | Immediate improvement in muscle strength and immediate alleviation of bulbar palsy and respiratory symptoms after a single exchange. | MG: 18 |
| **Dada 2004** | GBS Disability Grading Scale | A decrease of ≥1 grade at 4 weeks after TPE is defined as clinical improvement. | GBS: 10 |
| **Gafoor 2015** | Core clinical symptom alleviation | Treatment completion: significant symptom alleviation after only <5 exchanges is directly judged as treatment response. | Various neuroimmunological diseases |
| **Błądzińska 2020** | Core clinical symptom alleviation | Degree of improvement in limb muscle strength and respiratory function and alleviation of bulbar palsy and autonomic symptoms. | Various neuroimmunological diseases |
| **Kaya 2013** | Core clinical symptom alleviation | Complete improvement: complete resolution of neurological symptoms | Various neuroimmunological diseases |
|  |  | Partial improvement: marked relief of core symptoms including limb weakness, bulbar palsy, respiration, and walking |  |
|  |  | No response: no alleviation or even worsening of symptoms |  |
| **Kaynar 2008** | HFGS (0–6) | A decrease in grade when comparing scores before TPE and on the first day after completing the treatment course is defined as treatment response. | Various neuroimmunological diseases |
| **Korkmaz 2025** | Overall improvement on neurological examination | Complete remission (CR): complete disappearance of neurological deficit symptoms | Various neuroimmunological diseases |
|  |  | Partial remission (PR): marked alleviation but not complete resolution |  |
|  |  | No response (NR): no alleviation in neurological symptoms |  |
| **Kumar 2015** | MGFA clinical classification | Immediate improvement in muscle strength and immediate alleviation of bulbar palsy and respiratory symptoms after a single exchange. | MG: 35 |
| **Kumawat 2024** | Pagano 1–3 clinical severity grading | (I = marked improvement, II = mild improvement) | SPS: 5 |
|  |  | Based on the degree of relief of systemic symptoms including muscle stiffness, spasm, gait, balance, speech, and vision |  |
| **Lehmann 2008** | mRS | A decrease of ≥1 point in at least two of the three scores is defined as treatment response. | MMN: 7 |
|  | Upper/Lower Limb Disability Scales (ULDS/LLDS) |  |  |
|  | MRC sum score |  |  |
| **Corriveau 2023** | Subjective clinical efficacy stratification | Recorded by clinicians and classified into three categories: alleviated, unchanged, worsened | SPS: 39 |
|  | mRS | The mRS scores before TPE and at 3 months after treatment are compared. A decrease indicates functional improvement. |  |
| **Moser 2019** | Magaña grading criteria | No improvement: no improvement or even worsening | Various neuroimmunological diseases |
|  |  | Mild improvement: neurological symptoms reduced but no benefit in daily function |  |
|  |  | Marked improvement: neurological function recovered and daily activity ability significantly improved |  |
|  |  | Overall effective = mild + marked improvement |  |
| **Naik 2021** | mRS | mRS ≤2: good prognosis/treatment response, mRS ≥3: poor prognosis | AE: 4 |
| **Aristizábal 2020** | mRS | A decrease in mRS score after treatment | Various neuroimmunological diseases |
| **Pagano 2014** | 1–3 clinical improvement grading scale | Clinical improvement score: Grade 1: no symptom alleviation | SPS: 9 |
|  | Clinical improvement score | Grade 2: mild/partial improvement, no obvious change in daily activities |  |
|  |  | Grade 3: significant improvement, with recovery of gait, balance, speech, muscle spasm, etc. |  |
|  |  | Effective = Grade 2 + Grade 3 |  |
|  |  |  |  |
| **Seyhanli 2022** | GBS: HFGS | GBS: a decrease of ≥1 point after treatment is defined as treatment response. |  |
|  | CIDP: INCAT disability scale | CIDP: a decrease in score indicates functional improvement |  |
|  | MS/NMOSD/ADEM: EDSS | MS/NMOSD/ADEM: a decrease in score after treatment is defined as treatment response. |  |
|  | MG: MGC | MG: a decrease in score indicates symptom relief. |  |
| **Shah 2020** | Likert scale | Score ≥2 (mild, moderate, or marked improvement all classified as treatment response) | Various neuroimmunological diseases |
| **Sheckley 2021** | MGC | ① MGC: a decrease of ≥3 points after treatment is defined as clinically meaningful improvement. | MG: 14 |
|  | MG‑Activities of Daily Living (MG‑ADL) | ② MG-ADL: a decrease of ≥2 points after treatment is defined as clinically meaningful improvement. |  |
| **Sinanović 2017** | EDSS | EDSS >7 is defined as severe cases; a decrease in score after treatment indicates improvement. | Various neuroimmunological diseases |
| **Tombak 2016** | Comparison of neurological deficit symptoms before and after exchange | Complete remission (CR): complete disappearance of all neurological deficit symptoms | Various neuroimmunological diseases |
|  |  | Partial remission (PR): marked alleviation but not complete resolution |  |
|  |  | No response (NR): no alleviation in neurological symptoms |  |
|  |  | overall response rate = CR + PR |  |
| **Yamada 2015** | Subjective criteria for clinical symptom alleviation | Dysarthria, dysphagia, limb weakness, ptosis, diplopia, respiratory effort, fatigue | MG: 3 |
| **Yıldırım 2021** | GBS children: GBS disability score | Compare scores before TPE, at 2–4 weeks after exchange, and at last follow‑up | Various neuroimmunological diseases |
|  | All other children: pediatric mRS | A decrease ≥3 points: marked improvement |  |
|  |  | A decrease of 2 points: moderate improvement |  |
|  |  | A decrease of 1 point: mild improvement |  |
|  |  | No decrease: no improvement |  |
| **Zhang 2021** | mRS | Compare mRS scores at baseline, 1 month, 2 months, 6 months, and 12 months | AE: 33 |
|  |  | Clinical improvement criteria (any one): ① mRS score decreases by at least 1 point; ② disturbance of consciousness resolved; ③ weaned from ventilator; ④ seizures completely controlled. |  |
| **Totzeck 2022** | QMG | QMG: the decrease in QMG score after treatment is used as the objective efficacy evidence. | MG: 20 |
|  | Clinical subjective efficacy grading | Clinical subjective efficacy grading: effective: marked alleviation in weakness, bulbar, and respiratory symptoms |  |
|  |  | Partial improvement: symptoms reduced but still ventilator‑dependent |  |
|  |  | Ineffective: no relief or even worsening |  |
| **Fateen 2023** | Not reported | Not reported | Various neuroimmunological diseases |
| **Olsen 2021** | Symptom self‑assessment | Efficacy stratification: effective: symptom relief/disease progression slowed | TS‑HDS neuropathy: 17 |
|  |  | Ineffective: no change in neurological symptoms (5/17). |  |
| **Codron 2017** | Hughes neurological functional grading scale | Hughes: scored before TPE and at least 3 weeks after initiating exchange; a decrease in score indicates neurological improvement; grades 0 (asymptomatic) to 6 (death). | CDPN: 30 |
| **Vikrant 2017** | GBS Disability Grading Scale | ① GBS Disability Grading Scale: 0 (healthy) to 6 (death), comparing decrease from admission to discharge | GBS: 31 |
|  | MRC muscle strength grading | ② MRC muscle strength: upper and lower limb muscle strength scores, comparing improvement before and after treatment |  |
| **Pham 2025** | mRS | Clinical improvement criteria (any one): | AE: 36 |
|  |  | mRS score decreases by at least 1 point |  |
|  | Subjective criteria for clinical symptom alleviation | Improvement in consciousness or disturbance of consciousness resolved |  |
|  |  | Marked reduction in seizure frequency or severity |  |
| **Jamal 2025** | Not reported | Not reported | Various neuroimmunological diseases |
| **Gupta 2025** | mRS | A decrease in mRS score after treatment is defined as clinical improvement. | Various neuroimmunological diseases |
| **Eichinger 2025** | mRS | A score ≥2 is defined as clinical improvement. | Various neuroimmunological diseases |
|  | EDSS |  |  |
|  | Self‑developed 4‑level clinical efficacy score |  |  |

**Figure S1.** Forest plot of mortality rate

**Figure S2.** Sensitivity analysis, funnel plot and trim-and-fill analysis for overall response rate

**Figure S3.** Sensitivity analysis, funnel plot and trim-and-fill method of mortality

**Figure S4.** Overall response rate pooled across heterogeneous individual studies
